# Supplementary material for: Nasal Gene Expression in ART-Naive Adults with HIV and Pulmonary Tuberculosis in Uganda
Source: medRxiv. 2026 Jan 8:2026.01.06.26343354. Preprint. [Version 1] doi: 10.64898/2026.01.06.26343354 (PMC12803297; doi:10.64898/2026.01.06.26343354)
Supplement: Supplementary file 1 [file media-1.docx]

**Supplementary methods** 

**RNA sequencing and quality control**

Total cellular RNA from nasal and PAXgene blood samples was extracted using the RNeasy Plus Micro Kit protocol (QIAGEN Inc., Hilden, Germany) at Rutgers University according to the manufacturer’s instructions. The integrity of RNA was analyzed using an Agilent TapeStation. To generate the cDNA library, the NEBNext® rRNA Depletion Kit was used to remove the ribosomal RNA. Illumina-compatible RNA-seq library was then prepared using the NEB Next Ultra II RNASeq library preparation kit. The cDNA libraries were purified with AmpureXP beads and quantified using an Agilent TapeStation and a Qubit 4 Fluorometer.  Equimolar amounts of barcoded libraries were pooled and sequenced on the Illumina NovaSeq S2 FC 6000 (Illumina, San Diego, CA), producing 40-50 million paired-end 150 base-pair reads per sample. All samples were run across two lanes in a single flow cell in the same sequencing run.

Raw FASTQ data were assessed for quality using FastQC^1^. Trimmomatic was used to remove low quality bases and adaptors (LEADING:3 TRAILING:3 SLIDINGWINDOW:4:15 MINLEN:36)^2^. Reads were aligned to the human genome (GRCh38.p14) using the Spliced Transcripts Alignment to a Reference (STAR) tool^3^ and annotated using the GENCODE v44 primary assembly.^4^ 

Gene count data were exported to R (version 4.3.1) and subset for protein coding genes using the biomaRt package^5^.  Ensembl gene IDs were converted to gene names. Mitochondrial, hemoglobin, ribosomal and RNA genes, as well as low-count genes, were filtered out. Outliers were identified by examining the Euclidean distances between pairwise combinations of samples, visualized using ComplexHeatmap^6^ and principal component analysis (PCA). Tuberculosis (TB) status, age, sex, CD4 count, and HIV stage were investigated as potential expression-associated variables using PCA and corrected if needed (excluding TB status) using ComBat-Seq from the sva package^7^ for downstream analyzes. Differences in principal components for variables separating TB and controls were statistically tested using the Student’s t-test, given normality of data distribution.  All PCA and heatmap analyses were performed on transformed normalized counts (variance stabilizing transformation). 

## **Differential gene expression, machine learning and ROC analysis**

Differential gene expression analysis between adults with TB (HIV+/TB+)  and controls (HIV+/TB-) was performed using DESeq2^8^ in the nasal and blood datasets. DEGs were defined as having an adjusted p-value of <0.05 and log2 fold-change ≥1. Identified DEGs were used to cluster samples, visualized with ComplexHeatmap^6^, using Euclidean distance as a measure of dissimilarity and complete linkage for between-cluster separation.  Gene set enrichment analysis (GSEA) was performed using fgsea^9^ to evaluate whether DEGs from one tissue were enriched in the ranked list of genes from the other tissue. Genes were ranked based on the test statistic from DESeq2^8^. 

DEGs were used for training six machine learning models using caret^10^: random forest (RF),  support vector machine (SVM) with radial and linear kernels, elastic net, partial least squares (PLS) discriminant analysis and  k-nearest neighbors (KNN).  These models incorporate both linear and non-linear data relationships and are commonly used in TB diagnostic transcriptomic research^11-15^. Prior to model training, gene counts of nasal and blood DEGs underwent variance stabilizing transformation and predictors were pre-processed by removing near zero-variance variables. Centering and scaling for models sensitive to feature scaling (elastic net, SVM, KNN and PLS) were also performed. Receiver operating characteristic (ROC) curves for each model in diagnosing TB were constructed with the MLeval package^16^ to estimate the area under the curve (AUC), sensitivity, and specificity with 95% confidence intervals. Cut-off thresholds were determined by the maximum Youden index^17^.

Models with a reduced gene panel are potentially easier to translate to a diagnostic test. Variable importance for each nasal model trained using all DEGs in predicting TB status was estimated using caret^10^.  For the RF model, variable importance was performed based on permutation. The top 10 predictive genes for each model were identified and genes common to all six nasal models were retained to refit a new model using only these genes. The AUC, sensitivity and specificity of this parsimonious nasal signature in classifying TB was calculated using MLeval^16^. 

Finally, the INTERFEROME database (https://interferome.org/interferome/)  was used  to identify which top DEGs were interferon-stimulated genes, which we defined as genes that were significantly up or down regulated (>2.0-fold change, p<0.05) in human samples treated with interferon versus controls.

## **Pathway analysis and sample deconvolution** 

Pathway analyses for nasal and blood samples were performed with GSEA using the fgsea package^9^, and over-representation analysis (ORA) using the clusterProfiler package^18^.  For fGSEA, ranked gene lists using the test statistic were compared to Hallmark gene sets and KEGG and Reactome pathways from the Molecular Signatures Database (MSigDB)^19^. For ORA, gene ontology biological processes^20^ (GO BP) were evaluated in the differentially expressed upregulated and downregulated genes separately in adults with TB.  Pathways with adjusted p-value < 0.05 were considered functionally enriched, except for the nasal ORA analysis where no pathway met this threshold, therefore a threshold of adjusted p-value < 0.1 was applied. 

Nasal and blood gene lists from DESeq2 were also uploaded to the Qiagen’s Ingenuity Pathway Analysis (IPA) tool^21^ and filtered by adjusted p-value (false discovery rate) < 0.1 to determine canonical pathways and upstream regulators in TB. IPA uses a Fisher’s exact test to compute p-values and uses the log2 fold-change to compute z-scores.  For visualization, canonical pathways and upstream regulators with a p-value <0.05 and IPA z-score≥ ±1.5 were shown. 

Nasal samples consist of both immune and epithelial cell components. The CIBERSORTX  tool and the validated LM22 gene signature matrix^22,23^ were used to examine the immune cell composition in nasal and blood samples. CIBERSORTX is a deconvolution tool that employs support vector regression to estimate the relative proportion of immune cell subtypes from mixed bulk tissue transcriptome profiles^21^. The LM22 matrix consists of 547 genes that can accurately distinguish 22 immune cell populations, which can further be summarized into 11 major cell types based on shared lineage, including B cells, CD4 cells, CD8 cells, gamma delta T cells, eosinophils, plasma cells, NK cells, macrophages, neutrophils, dendritic cells and mast cells^22^. Compared to other deconvolution methods, CIBERSORT has been shown to be more robust and accurate in enumerating cell composition of complex tissues, including those with low immune cell content and high levels of noise.^20^ The LM22 signature has also been widely used to explore immune cell composition in airway samples where immune cells are not predominant^24-26^.

Counts data were first normalized as transcripts per million (TPM) to match the signature matrix. CIBERSORTX was performed with B-mode batch correction, quantile normalization disabled, and the number of permutations set to 500. Results were expressed as fractions of total leukocyte content. Proportions of estimated immune cell populations were compared by TB status using either Student’s t-test or Mann-Whitney U test depending on normality of data distribution, with analyses corrected for multiple comparisons. 

## **Performance of published blood RNA signatures using the TBSignatureProfiler**

The TBSignatureProfiler package^27^ was used to apply published blood signatures that diagnose TB disease to our nasal and blood RNA sequencing datasets. We selected blood signatures that were shown to distinguish TB from latent TB infection, TB from some combination of other diseases or TB from HIV. Raw counts were first converted into log counts per million (CPM). Gene set variation analysis (GSVA) and single-sample GSEA (ssGSEA) scoring methods were used to estimate the diagnostic performance of 59 published blood signatures. Signature scores were compared using two-sample t-tests and AUC values, along with confidence intervals derived from bootstrapping. Visualization was done using boxplots. Only published blood signatures with two or more genes that coincided with genes in our sequencing data were used in the analysis. 

## **Computational code** 

Computational code for the methodology is available at: https://github.com/nisreenkhambati/uganda_nasal_cells

**References for Supplementary Methods**

1.	Andrews S. FastQC:  A Quality Control Tool for High Throughput Sequence Data 2010 [Available from: [http://www.bioinformatics.babraham.ac.uk/projects/fastqc/](http://www.bioinformatics.babraham.ac.uk/projects/fastqc/).

2.	Bolger AM, Lohse M, Usadel B. Trimmomatic: a flexible trimmer for Illumina sequence data. Bioinformatics. 2014;30(15):2114-20. doi:10.1093/bioinformatics/btu170

3.	Dobin A, Davis CA, Schlesinger F, Drenkow J, Zaleski C, Jha S, et al. STAR: ultrafast universal RNA-seq aligner. Bioinformatics. 2013;29(1):15-21. doi:10.1093/bioinformatics/bts635

4.	GENCODE. Human Release 44 (GRCh38.p14) 2022 [Available from: [https://www.gencodegenes.org/human/release_44.html](https://www.gencodegenes.org/human/release_44.html).

5.	Durinck S, Spellman PT, Birney E, Huber W. Mapping identifiers for the integration of genomic datasets with the R/Bioconductor package biomaRt. Nat Protoc. 2009;4(8):1184-91. doi:10.1038/nprot.2009.97

6.	Gu Z, Eils R, Schlesner M. Complex heatmaps reveal patterns and correlations in multidimensional genomic data. Bioinformatics. 2016;32(18):2847-9. doi:10.1093/bioinformatics/btw313

7.	Zhang Y, Parmigiani G, Johnson WE. ComBat-seq: batch effect adjustment for RNA-seq count data. NAR Genom Bioinform. 2020;2(3):lqaa078. doi:10.1093/nargab/lqaa078

8.	Love MI, Huber W, Anders S. Moderated estimation of fold change and dispersion for RNA-seq data with DESeq2. Genome Biol. 2014;15(12):550. doi:10.1186/s13059-014-0550-8

9.	Korotkevich G, Sukhov V, Sergushichev A. Fast gene set enrichment analysis. bioRxiv. 2019. 

10.	Kuhn M. Building Predictive Models in R Using the caret Package. Journal of Statistical Software. 2008;28(5):1 - 26. doi:10.18637/jss.v028.i05

11.	Channon-Wells S, Habgood-Coote D, Vito O, Galassini R, Wright VJ, Brent AJ, et al. Integration and validation of host transcript signatures, including a novel 3-transcript tuberculosis signature, to enable one-step multiclass diagnosis of childhood febrile disease. J Transl Med. 2024;22(1):802. doi:10.1186/s12967-024-05241-4

12.	Bobak CA, Titus AJ, Hill JE. Comparison of common machine learning models for classification of tuberculosis using transcriptional biomarkers from integrated datasets. Applied Soft Computing. 2019;74:264-73. doi:[https://doi.org/10.1016/j.asoc.2018.10.005](https://doi.org/10.1016/j.asoc.2018.10.005)

13.	Anderson ST, Kaforou M, Brent AJ, Wright VJ, Banwell CM, Chagaluka G, et al. Diagnosis of childhood tuberculosis and host RNA expression in Africa. N Engl J Med. 2014;370(18):1712-23. doi:10.1056/NEJMoa1303657

14.	Ding S, Yi X, Gao J, Huang C, Zhou Y, Yang Y, et al. Combining bioinformatics and machine learning to identify diagnostic biomarkers of TB associated with immune cell infiltration. Tuberculosis (Edinb). 2024;149:102570. doi:10.1016/j.tube.2024.102570

15.	Turner CT, Gupta RK, Tsaliki E, Roe JK, Mondal P, Nyawo GR, et al. Blood transcriptional biomarkers for active pulmonary tuberculosis in a high-burden setting: a prospective, observational, diagnostic accuracy study. Lancet Respir Med. 2020;8(4):407-19. doi:10.1016/S2213-2600(19)30469-2

16.	John CR. MLeval: Machine Learning Model Evaluation 2020 [Computer software version 0.3]. Available from: [https://cran.r-project.org/web/packages/MLeval/index.html](https://cran.r-project.org/web/packages/MLeval/index.html).

17.	Fluss R, Faraggi D, Reiser B. Estimation of the Youden Index and its associated cutoff point. Biom J. 2005;47(4):458-72. doi:10.1002/bimj.200410135

18.	Wu T, Hu E, Xu S, Chen M, Guo P, Dai Z, et al. clusterProfiler 4.0: A universal enrichment tool for interpreting omics data. Innovation (Camb). 2021;2(3):100141. doi:10.1016/j.xinn.2021.100141

19.	Broad Institute. Human MSigDB Collections: Broad Institute, Inc., Massachusetts Institute of Technology, and Regents of the University of California; 2023 [Available from: [https://www.gsea-msigdb.org/gsea/msigdb/collections.jsp](https://www.gsea-msigdb.org/gsea/msigdb/collections.jsp).

20.	Gene Ontology C, Aleksander SA, Balhoff J, Carbon S, Cherry JM, Drabkin HJ, et al. The Gene Ontology knowledgebase in 2023. Genetics. 2023;224(1). doi:10.1093/genetics/iyad031

21.	Kramer A, Green J, Pollard J, Jr., Tugendreich S. Causal analysis approaches in Ingenuity Pathway Analysis. Bioinformatics. 2014;30(4):523-30. doi:10.1093/bioinformatics/btt703

22.	Newman AM, Liu CL, Green MR, Gentles AJ, Feng W, Xu Y, et al. Robust enumeration of cell subsets from tissue expression profiles. Nat Methods. 2015;12(5):453-7. doi:10.1038/nmeth.3337

23.	Newman AM, Steen CB, Liu CL, Gentles AJ, Chaudhuri AA, Scherer F, et al. Determining cell type abundance and expression from bulk tissues with digital cytometry. Nat Biotechnol. 2019;37(7):773-82. doi:10.1038/s41587-019-0114-2

24.	Hurst JH, Mohan AA, Dalapati T, George IA, Aquino JN, Lugo DJ, et al. Differential host responses within the upper respiratory tract and peripheral blood of children and adults with SARS-CoV-2 infection. medRxiv. 2023:2023.07.31.23293337. doi:10.1101/2023.07.31.23293337

25.	Kan X, Guan R, Hao J, Zhao C, Sun Y. Integrative analysis of immune-related signature profiles in eosinophilic chronic rhinosinusitis with nasal polyposis. FEBS Open Bio. 2023;13(12):2273-89. doi:10.1002/2211-5463.13720

26.	Xu F, Vasilescu DM, Kinose D, Tanabe N, Ng KW, Coxson HO, et al. The molecular and cellular mechanisms associated with the destruction of terminal bronchioles in COPD. Eur Respir J. 2022;59(5). doi:10.1183/13993003.01411-2021

27.	Johnson WE, Odom A, Cintron C, Muthaiah M, Knudsen S, Joseph N, et al. Comparing tuberculosis gene signatures in malnourished individuals using the TBSignatureProfiler. BMC Infect Dis. 2021;21(1):106. doi:10.1186/s12879-020-05598-z
